# Novel anti-tumour necrosis factor receptor-1 (TNFR1) domain antibody prevents pulmonary inflammation in experimental acute lung injury

**DOI:** 10.1136/thoraxjnl-2017-210305

**Published:** 2018-01-29

**Authors:** Alastair Proudfoot, Andrew Bayliffe, Cecilia M O’Kane, Tracey Wright, Adrian Serone, Philippe Jean Bareille, Vanessa Brown, Umar I Hamid, Younan Chen, Robert Wilson, Joanna Cordy, Peter Morley, Ruud de Wildt, Stuart Elborn, Matthew Hind, Edwin R Chilvers, Mark Griffiths, Charlotte Summers, Daniel Francis McAuley

**Affiliations:** 1 National Heart and Lung Institute, Imperial College, London, UK; 2 GlaxoSmithKline Research and Development, Stevenage, UK; 3 School of Medicine, Dentistry and Biomedical Sciences, Centre for Experimental Medicine, Queen’s University of Belfast, Belfast, UK; 4 GlaxoSmithKline R&D, Philadelphia, Pennsylvania, USA; 5 National Institute for Health Research Respiratory Biomedical Research Unit, Royal Brompton and Harefield NHS Foundation Trust, London, UK; 6 Department of Medicine, University of Cambridge School of Clinical Medicine, Cambridge, UK

**Keywords:** ARDS, neutrophil biology

## Abstract

**Background:**

Tumour necrosis factor alpha (TNF-α) is a pleiotropic cytokine with both injurious and protective functions, which are thought to diverge at the level of its two cell surface receptors, TNFR1 and TNFR2. In the setting of acute injury, selective inhibition of TNFR1 is predicted to attenuate the cell death and inflammation associated with TNF-α, while sparing or potentiating the protective effects of TNFR2 signalling. We developed a potent and selective antagonist of TNFR1 (GSK1995057) using a novel domain antibody (dAb) therapeutic and assessed its efficacy in vitro, in vivo and in a clinical trial involving healthy human subjects.

**Methods:**

We investigated the in vitro effects of GSK1995057 on human pulmonary microvascular endothelial cells (HMVEC-L) and then assessed the effects of pretreatment with nebulised GSK1995057 in a non-human primate model of acute lung injury. We then tested translation to humans by investigating the effects of a single nebulised dose of GSK1995057 in healthy humans (n=37) in a randomised controlled clinical trial in which subjects were subsequently exposed to inhaled endotoxin.

**Results:**

Selective inhibition of TNFR1 signalling potently inhibited cytokine and neutrophil adhesion molecule expression in activated HMVEC-L monolayers in vitro (P<0.01 and P<0.001, respectively), and also significantly attenuated inflammation and signs of lung injury in non-human primates (P<0.01 in all cases). In a randomised, placebo-controlled trial of nebulised GSK1995057 in 37 healthy humans challenged with a low dose of inhaled endotoxin, treatment with GSK1995057 attenuated pulmonary neutrophilia, inflammatory cytokine release (P<0.01 in all cases) and signs of endothelial injury (P<0.05) in bronchoalveolar lavage and serum samples.

**Conclusion:**

These data support the potential for pulmonary delivery of a selective TNFR1 dAb as a novel therapeutic approach for the prevention of acute respiratory distress syndrome.

**Trial registration number:**

ClinicalTrials.gov NCT01587807.

**Key messages**

**What is the key question?**

- Does selectively targeting tumour necrosis factor receptor 1 (TNFR1) signalling with a novel domain antibody (dAb) reduce pulmonary inflammation?

**What is the bottom line?**

- A dAb selective to the TNFR1 reduced pulmonary inflammation in both non-human primate and human pulmonary endotoxin challenge models when dosed prophylactically. Data from mechanistic studies in human cells with this dAb support the importance of TNFR1 signalling in mediating endothelial–neutrophil interactions associated with the development of lung injury.

**Why read on?**

- This investigation provides original data to support the utility of a novel TNFR1-targeting dAb as a potential therapy in patients with acute respiratory distress syndrome and supports further clinical investigation in patients.

## Introduction

Acute respiratory distress syndrome (ARDS) affects approximately 190 000 patients per annum in the USA, and is associated with mortality rates of up to 40%.^1^ To date, no effective pharmacological therapies, which target the underlying pathophysiological mechanisms of ARDS, have been discovered.^2^ The development of ARDS is characterised by immune cell-mediated injury to the lung,^3 4^ associated with the release of inflammatory cytokines and proteases. The uncontrolled local inflammatory response in ARDS contributes to alveolar-capillary barrier damage and the exudation of protein-rich fluid into the alveolar space, which manifests as non-cardiogenic pulmonary oedema.^5^ Pulmonary neutrophil recruitment, which is central to the pathogenesis of ARDS, is mediated by the interaction of primed and activated neutrophils with the lung microvascular endothelium.^6^ Preclinical and clinical studies have identified tumour necrosis factor alpha (TNF-α) as a key effector molecule in ARDS and also sepsis; a common cause of ARDS.^7 8^ However, results from clinical trials of parenteral non-selective TNF-α targeting antibodies in sepsis have been variable with most failing to demonstrate a significant survival benefit^9–12^; at least one trial suggesting evidence of harm at higher doses^13^; and one trial suggesting an improvement in hospital survival and organ dysfunction in patients with elevated baseline interleukin-6 (IL-6) levels.^14^

Recently, it has become evident that the pleiotropic effects of TNF-α diverge at the level of its two cellular receptors, TNF receptor 1 (TNFR1) and TNF receptor 2 (TNFR2).^15^ Whereas numerous studies support the role of TNFR1 in mediating cell death signalling and inflammation,^16^ the role of TNFR1 in the generation of vascular leak and neutrophilic inflammation remains controversial.^17 18^ In contrast, it has recently been suggested that TNFR2 signalling may be important in attenuating the apoptotic activity of TNF-α, and in mediating important survival and proliferation signals^19–21^ through activation of apoptotic regulators (eg, cIAP1 and 2) and the non-canonical nuclear factor κB pathway, respectively.^22^ Interestingly, TNFR1-deficient mice are protected from lung injury, sepsis and other acute organ injuries, whereas TNFR2-deficient mice are consistently more susceptible to injury in these models.^20 23–26^ These studies indicate that selectively antagonising TNFR1, while sparing TNFR2 signalling, could be therapeutically advantageous. We therefore developed a short-acting, fully human domain antibody (dAb) fragment that selectively antagonises TNF-α signalling through TNFR1, but not TNFR2.

dAbs represent the smallest functional antigen-binding units of human antibodies (10–13 kDa), are derived from single variable regions of either heavy or light chain sequences of human IgG and are sufficiently stable to allow nebulisation directly to the lungs.^27^ A homology model structure and amino acid sequence of GSK1995057 are shown in online supplementary figure 1, together with binding characteristics to both human and cynomolgus monkey TNFR1-Fc fusion proteins. When administered directly to the lungs of mice either concomitantly or after challenge with acid instillation or ventilation with large tidal volumes, murine TNFR1-targeting dAbs attenuate the development of pulmonary oedema, arterial hypoxaemia and inflammation.^28 29^ Here we present the first report of an inhaled variable heavy (V_H_) chain TNFR1 dAb antagonist (GSK1995057) administered directly to the lungs of cynomolgus monkeys and humans, and explore the underlying mechanism of action using in vitro human tissue assays.

10.1136/thoraxjnl-2017-210305.supp1 Supplementary file 1

## Methods

### In vitro studies in human cells

The assessment of permeability of human pulmonary microvascular endothelial cell (HMVEC-L) monolayers was undertaken using an electrical cell impedance sensor technique. Once electrical resistance achieved steady state, cells were incubated for 1 hour in the presence of GSK1995057 or dummy dAb (10 nM) or vehicle control for 1 hour prior to stimulation with TNF-α (10 ng/mL). Neutrophil transmigration assays were performed using neutrophils isolated from healthy volunteers. Neutrophil transmigration through confluent HMVEC-L incubated with GSK1995057 or dummy dAb (10 nM) or vehicle control for 1 hour prior to stimulation with TNF-α (10 ng/mL) was measured using CytoSelect Leukocyte Transmigration assay (Cambridge Biosciences) and endothelial ligand cell surface expression was assessed using HMVEC-L after 4 hours of treatment. Detailed descriptions of assay conditions are contained in the online supplementary data. Samples were analysed on a FACSCanto II flow cytometer (BD Biosciences) as described in the online supplementary data. In all in vitro experiments with GSK1995057 were compared with a non-targeting ‘dummy dAb’ comprising natural human V_H_ framework sequences and ‘scrambled’/non-sense hypervariable domains.

### In vivo study in cynomolgus monkeys

The objective of this study was to investigate the dose–response relationship of single doses of GSK1995057 given as a pretreatment in a lipopolysaccharide (LPS)-induced model of pulmonary inflammation in sedated young adult cynomolgus monkeys. The primary endpoint was neutrophil counts in bronchoalveolar lavage fluid (BALF) at 6 and 24 hours after dosing.

#### Animal husbandry

Animals were housed according to the USDA Animal Welfare Act (9 CFR, parts 1, 2 and 3) and as described in the Guide for the Care and Use of Laboratory Animals.^30^ Temperatures of 18°C–29°C (64°F–84°F) with a relative humidity of 50%±20% were maintained, along with 10 or greater air changes per hour with 100% fresh air (no air recirculation), and a 12-hour light/12-hour dark cycle. LabDiet Certified Primate Diet 5048 was fed to the animals daily, supplemented with washed, fresh produce. Before anaesthesia, animals were fasted for a minimum of 4 hours. Reverse-osmosis, postchlorinated water was provided ad libitum, including during times of fasting.

#### Study procedures

A description of the methodology for administration of anaesthesia and LPS is present in the online supplementary data. One hour after administration of GSK1995057, or vehicle, LPS *Escherichia coli* serotype 055:B5 (4 mL of 100 µg/mL) was administered via aerosolisation (DeVilbiss Ultraneb-99 ultrasonic nebuliser) over 5 min. Blood and bronchoalveolar lavage (BAL) samples were collected at baseline (before challenge), 6 and 24 hours after LPS challenge. Detailed descriptions of the techniques used for bronchoscopy and bronchoalveolar lavage are contained within the online supplementary data.

#### Biomarker assays

Cynomolgus monkey BAL samples were analysed by Myriad RBM using their Multi-Analyte Platform (MAP) technology on the Human MAPv1.6 panel of 89 biomarkers, 78 of which are confirmed to be cynomolgus monkey cross-reactive.

#### Study approval

All studies were conducted in accordance with the GSK Policy on Care, Welfare, and Treatment of Laboratory Animals, and were reviewed by the Institutional Animal Care and Use Committee at Charles River Laboratories.

### Clinical trial in healthy volunteers

#### Participants

Healthy subjects were recruited by advertising. Screening consisted of a history and physical examination, blood investigations, ECG and spirometry (full clinical trial protocol inclusion and exclusion criteria and study schedule are outlined in the data file and table E1, respectively, in the online supplementary data).

#### Study design

The clinical trial was a randomised, placebo-controlled study to investigate the safety, tolerability, pharmacokinetics and pharmacodynamics of single doses of inhaled GSK1995057 in healthy subjects. The study consisted of 2 parts within a ‘fused’ protocol operated across two different clinical units, recruiting a total of six cohorts. The dose-escalating cohorts in part 1 were conducted to confirm preliminary safety, tolerability and pharmacokinetics of GSK1995057 and were conducted at the PAREXEL International Clinical Pharmacology Research Unit, Harrow, UK. This part of the study was conducted in a single-blind manner to allow appropriate, real-time assessment of safety. Subjects in cohort 5 of part 1 received a single inhaled dose of GSK1995057 in addition to BAL sampling at approximately 30 min after dose to confirm BALF levels of GSK1995057.

Subjects in part 2 of the trial were randomised to receive a single nebulised dose (26 mg) of GSK1995057 1 hour prior to receiving a nebulised challenge of 50 µg of *E. coli* LPS. This part of the study was carried out at Celerion Clinical Pharmacology Unit, Belfast, UK. The BAL procedure was performed 6 hours after LPS inhalation (7 hours after dosing GSK1995057) and the primary endpoint of the trial was BALF neutrophil count with BALF and plasma cytokine, chemokine, epithelial and endothelial biomarkers as secondary endpoints. The dose of GSK1995057 and timing for BAL was derived from data obtained from the dose-finding study in cynomolgus monkeys also presented in this manuscript. A detailed description of the study design, administration of the study drug, bronchoscopy, BAL and sample collection are contained within the online supplementary data. Pharmacokinetic sampling was performed at varying time points up to 48 hours after the start of nebulisation of GSK1995057, and concentrations of GSK1995057 in plasma and BALF were measured by electrochemiluminescence immunoassay (ECLIA) on the MesoScale Discovery (MSD) platform (Gaithersburg, MD, USA) (lower limit of quantification=100 ng/mL).

#### Biomarker assays

The measurement of biomarkers in BALF and serum samples from the clinical trial participants were tested under contract by Myriad RBM (Austin, Texas, USA) using their proprietary multiplex Luminex immunoassay platform; the human inflammation multiplex MAP (iMAP). Biomarkers of interest not included on this panel were measured using commercial ELISAs (surfactant protein-D (SP-D) and Club cell secretory protein (CC16) ELISAs from BioVendor), following manufacturers’ recommendations under contract by Quotient BioResearch (Fordham, Cambridgeshire, UK). Changes from baseline in free and total TNFR1 were evaluated at varying time points up to 48 hours after the start of nebulisation using an ECLIA on the MSD platform and were conducted by GSK.

#### Study approval

The study was undertaken in accordance with the Declaration of Helsinki and ICH GCP. Written informed consent was obtained from all subjects prior to enrolment in the study.

#### Statistical analysis

**Figure 1:**
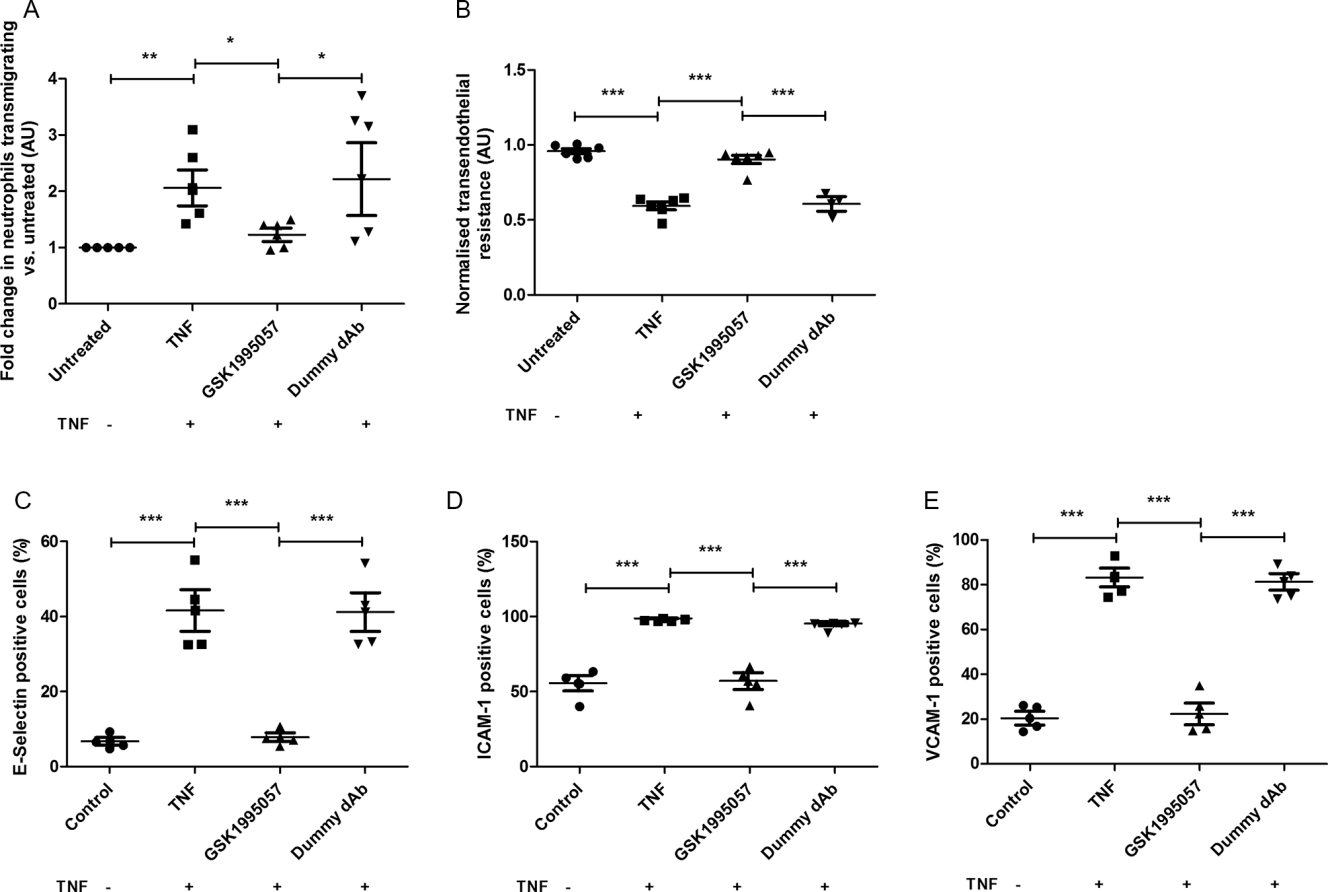
Effects of GSK1995057 on neutrophil–human pulmonary microvascular endothelial interactions in vitro. Panel (A) shows the effects of GSK1995057 on the transmigration of Leukotracker labelled human neutrophils towards interleukin (IL)-8, through TNF-α-treated human pulmonary microvascular endothelial cell monolayers. Panel (B) shows the effect of GSK1995057 on the transendothelial resistance, a marker of permeability, of TNF-α-treated human pulmonary microvascular monolayers. Panels (C–E) show the effect of GSK1995057 on the cell surface expression of the key neutrophil ligands E-selectin, ICAM-1 and VCAM-1 by TNF-α-treated human pulmonary microvascular monolayers. Data shown are geometric mean±SEM ((A) n*=*6; (B) n=6 (dummy dAb condition n=3); (C–E) n=4). Data analysed with analysis of variance (ANOVA) or Kruskal-Wallis rank-sum test (A). *P<0.05; **P<0.01; ***P<0.001. AU, arbitrary units; dAb, domain antibody; ICAM, intercellular adhesion molecule; TNF, tumour necrosis factor; VCAM, vascular cell adhesion molecule.

Analysis of non-clinical data from in vitro and in vivo studies with GSK1995057 was undertaken using analysis of variance on appropriately transformed data, with the exception of neutrophil migration data (figure 1A) and soluble E-selectin data (figure E2a in the online supplementary data) for which a non-parametric analysis (Kruskal-Wallis rank-sum test) was necessary. P<0.05 was accepted as a significant difference between comparisons. All data are presented as geometric mean and SE and no multiplicity adjustments were made.

The clinical trial was designed to recruit enough subjects to provide data from 36 subjects available for analysis (18 for placebo, and 18 for GSK1995057). While the sample size was based on feasibility, subject numbers were determined to ensure at least 80% power to detect a 65% decrease in BALF neutrophil counts based on mean effect sizes observed in monkey LPS challenge studies. An interim sample size re-estimation (blinded to investigator site team) was undertaken when BAL neutrophil counts from 16 subjects (eight in each study arm) were available for statistical analysis. This confirmed the estimates used to assess the statistical power of the planned sample size, and therefore no change to the sample size was made. Distributional assumptions were assessed by visual inspection of residual plots. Normality was examined by normal probability plots, while homogeneity of variance was assessed by plotting the residuals against the predicted values for the model. Two sample t-tests were used to evaluate the effects of GSK1995057 on biomarkers from BALF samples. Mixed model repeated measures using random effects was used to evaluate the effects of GSK1995057 on biomarkers from serum samples using SAS V.9.3. Baseline values of the biomarker were included as covariates in the analysis of biomarkers from serum samples. Data are shown as geometric mean and SE.

## Results

### TNFR1-mediated neutrophil–endothelial cell interactions in vitro

To investigate the hypothesis that TNFR1 mediates neutrophilic inflammation in the lung, we assessed the impact of selective TNFR1 antagonism using GSK1995057 on neutrophil transmigration and endothelial permeability in a human in vitro cell system. Using the CytoSelect Leukocyte Transmigration assay, we observed that pretreatment of HMVEC-L monolayers with GSK1995057 significantly reduced the transendothelial migration of neutrophils towards the neutrophil chemoattractant IL-8 (figure 1A; P<0.05). GSK1995057 also reduced TNF-α-mediated endothelial permeability (figure 1B; P<0.001), and modulated the expression of key ligands for neutrophil–endothelial interaction, preventing the TNF-α-induced increases in HMVEC-L surface expression of E-selectin, intercellular adhesion molecule (ICAM)-1 and vascular cell adhesion molecule (VCAM)-1 (P<0.001 in all cases; figure 1C–E), as well as decreasing the concentrations of the soluble forms of these molecules, which are markers of endothelial injury (P<0.05 in all cases; see online supplementary figure 2). TNFR1 antagonism also reduced the release of the cytokines IL-1β, IL-6 and IL-8 by TNF-α-stimulated HMVEC-L (P<0.01 in all cases; see online supplementary figure 3).

### TNFR1 inhibition with GSK1995057 prevented acute lung injury in a non-human primate model

**Figure 2:**
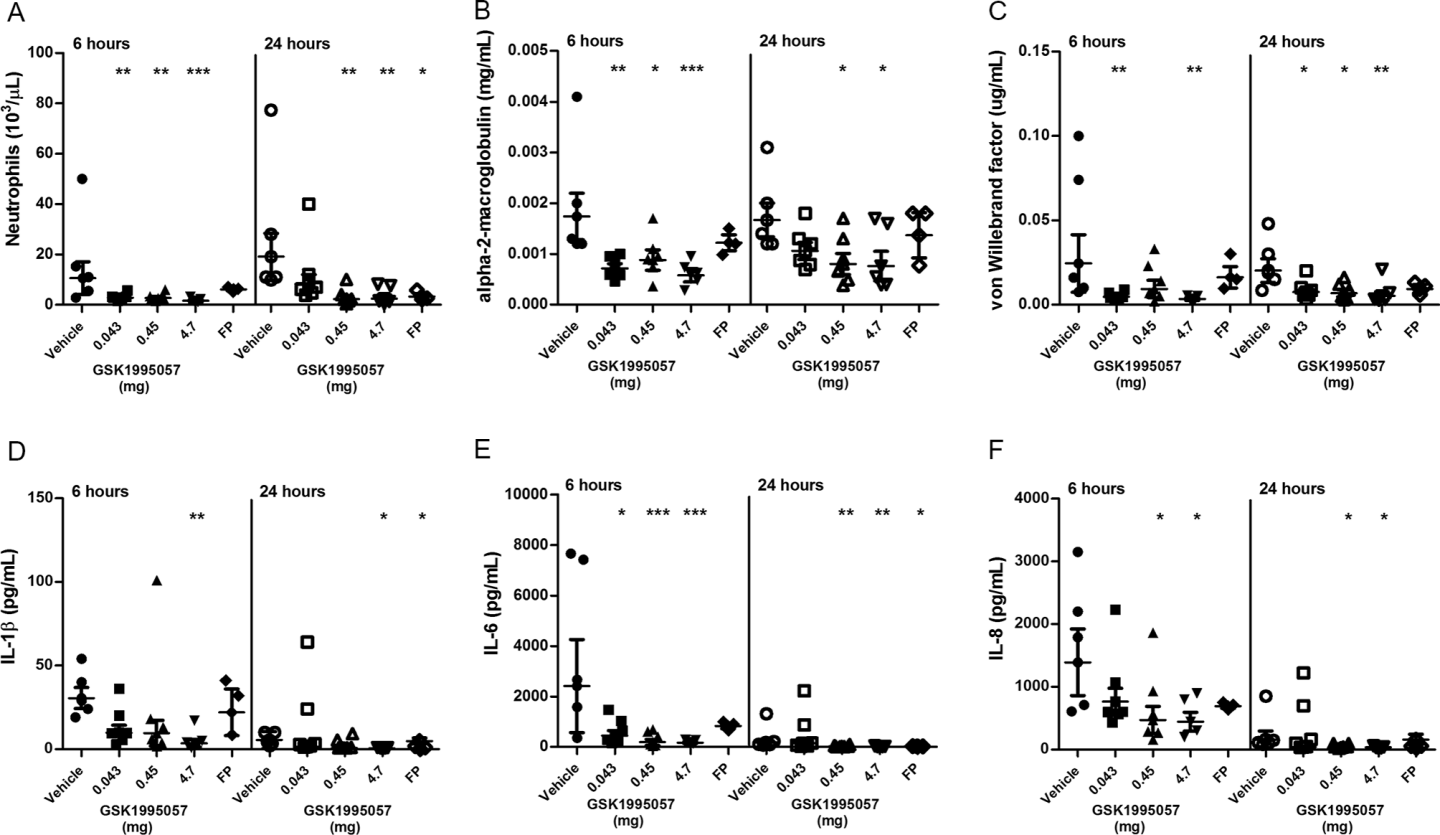
Effects of GSK1995057 in a non-human primate experimental model of acute lung injury. Cynomolgus monkeys received nebulised GSK1995057 (0.043, 0.45 or 4.7 mg), vehicle control, or an intratracheal instillation of fluticasone propionate (FP) (positive control; 1.2 mg), 1 hour prior to nebulised lipopolysaccharide (LPS; 4 mL of 100 µg/mL) challenge. Bronchoalveolar lavage fluid (BALF) was collected at 6 and 24 hours after LPS challenge. The effects of inhaled GSK1995057 (0.043, 0.45 or 4.7 mg dose) are shown on BALF neutrophil counts, and BALF concentrations of the alveolar-capillary permeability marker alpha-2-macroglobulin and the biomarker of endothelial activation/injury von Willebrand factor (A–C). Panels (D–F) show the effects of GSK1995057 on BALF concentrations of interleukin (IL)-1β, IL-6 and IL-8, respectively. Data shown are geometric means±SEM (n=3 monkeys in the FP group, n=5 in the 4.7 mg GSK1995057 and vehicle groups, and n=6 in the 0.45 and 0.043 mg GSK1995057 groups). Data analysed with analysis of variance (ANOVA). *P<0.05; **P<0.01; ***P<0.001.

We investigated further the effects of a single aerosolised dose of GSK1995057 on pulmonary inflammation in a non-human primate model of acute lung injury. Twenty-five cynomolgus monkeys received either nebulised vehicle control, nebulised GSK1995057 (0.043 mg, 0.45 mg or 4.7 mg) or an instillation of fluticasone propionate (positive control; 1.2 mg) via the intratracheal route, 1 hour prior to nebulised LPS (400 µg) challenge. Nebulised GSK1995057 was well tolerated in monkeys and no adverse effects associated with dosing were observed (data not shown). The primary endpoint was the number of neutrophils in the BALF at 6 and 24 hours after LPS exposure. BALF neutrophil counts were markedly suppressed at 6 hours for all three doses of GSK1995057, and at 24 hours for the higher dose levels (0.45 and 4.7 mg (P<0.01 in all cases; figure 2A)). In support of this finding, the BALF concentration of neutrophil-derived myeloperoxidase was also reduced in the GSK1995057-treated animals (see online supplementary figure 4A). A reduction in BALF monocyte-macrophage numbers was also observed at 24 hours, reaching significance with the highest and middle doses of GSK1995057 (P<0.01, online supplementary figure 4B). In agreement with our in vitro studies, pretreatment with GSK1995057 reduced the BALF concentration of alpha-2-macroglobulin, a marker of alveolar-capillary barrier permeability (figure 2B) and von Willebrand factor, a biomarker of endothelial injury/activation (vWF; figure 2C). Selective TNFR1 antagonism with GSK1995057 also reduced BALF concentrations of IL-1β, IL-6 and IL-8, compared with vehicle-treated animals (P<0.05 in all cases; figure 2D–F).

### TNFR1 inhibition with GSK1995057 prevented acute lung injury in a healthy human volunteer model

Having demonstrated that selective TNFR1 antagonism with GSK1995057 reduced neutrophilic pulmonary inflammation, microvascular permeability and endothelial cell activation/injury in complementary in vitro and in vivo models, we undertook a double-blinded, placebo-controlled clinical trial to determine the effects of nebulised GSK1995057 in an LPS-induced model of acute lung injury in healthy human volunteers. Previous clinical investigations with intravenous GSK1995057 identified a pre-existing host antibody response in some human subjects that increased the risk of mild infusion reactions following intravenous dosing.^31^ As a result, only healthy subjects prospectively demonstrated to be seronegative for these pre-existing antidrug antibodies were eligible for participation. Following a dose escalation study to determine the safety, tolerability, pharmacokinetics and target engagement of nebulised GSK1995057 (see online supplementary figures 5–7), healthy human subjects were randomised (1:1) to receive a single nebulised dose (26 mg) of GSK1995057 or placebo (see online supplementary table 2 for details of investigational products), administered 1 hour prior to the inhalation of LPS (50 µg), with BALF collected 6 hours after LPS challenge (ClinicalTrials.gov identifier NCT01587807). The LPS challenge schema is outlined in online supplementary figure 8, and the full study schedule is shown in online supplementary table 1. The primary endpoint of the study was the BALF neutrophil count 6 hours after LPS exposure (7 hours after dosing of GSK1995057).

**Figure 3:**
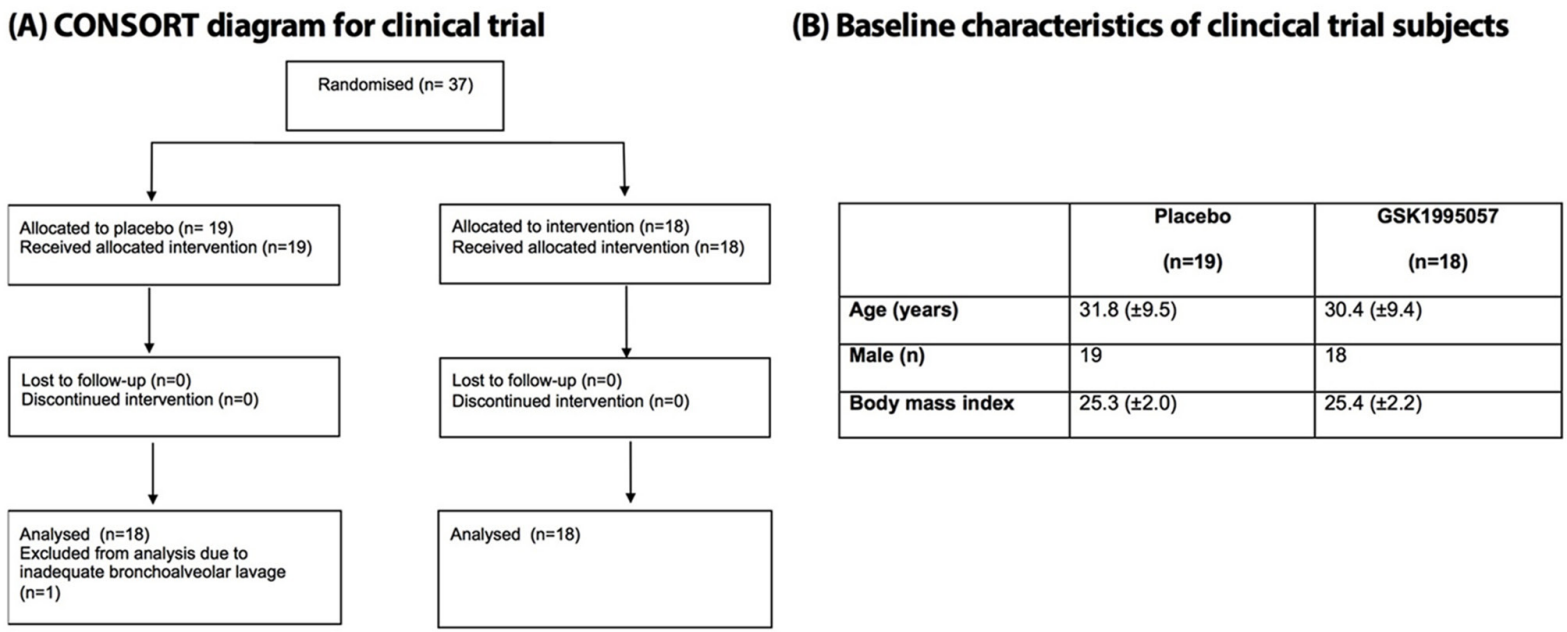
Consolidated Standards of Reporting Trials (CONSORT) diagram for clinical trial and baseline characteristics of study subjects. Panel (A) shows the CONSORT diagram for the double-blinded, placebo-controlled, clinical study of GSK1995057 in healthy volunteers exposed to inhaled lipopolysaccharide (LPS). Panel (B) details the baseline characteristics of the subjects recruited to the clinical study.

Thirty-seven healthy subjects were enrolled. One subject in the placebo group was excluded from cell count analyses, due to technical failure of automated cell counting. Since the sample was otherwise viable, protein biomarker results from this subject were included in the analyses, but an additional subject was recruited to the placebo group to provide additional cell count data. The Consolidated Standards of Reporting Trials diagram for the study is shown in figure 3A. There were no differences in baseline demographic parameters between the groups randomised to receive placebo or GSK1995057 (figure 3B). No serious or unexpected adverse events occurred in either group. The table of adverse events is shown in online supplementary table 3.

**Figure 4:**
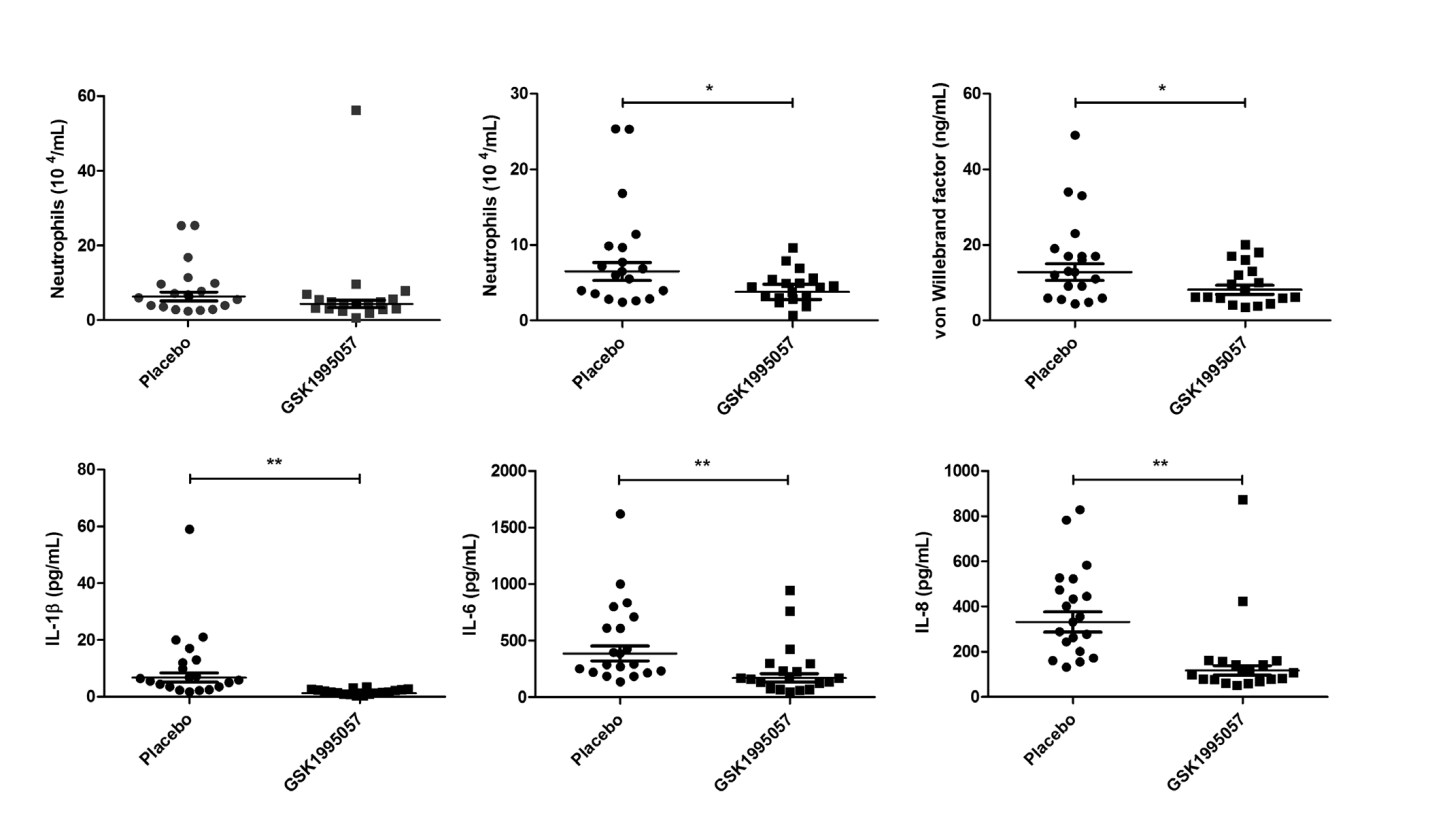
Effects of GSK1995057 in a human experimental model of acute lung injury. Healthy human subjects (n=37) were randomised (1:1) to treatment with GSK1995057, or vehicle control, 1 hour prior to inhalation of 50 µg lipopolysaccharide (LPS). Bronchoalveolar lavage fluid (BALF) was collected 6 hours after LPS inhalation. The effect of GSK1995057 on BALF neutrophil count is shown with (A) and without (B) a single biological outlier who displayed an exaggerated increase in BALF neutrophils (>3 times IQR, and outside the upper quartile). BALF cytology in this subject was abnormal with the presence of foamy macrophages, excessive mucus and debris, and lytic/apoptotic neutrophils; however, the subject was clinically well and had a transient increase in temperature (38.2°C) after LPS inhalation, but no clinically significant adverse events. Panel (C) shows the effect of GSK1995057 on BALF concentration of the endothelial activation/injury biomarker von Willebrand factor. Panels (D–F) show the effect of GSK1995057 on BALF concentrations of interleukin (IL)-1β, IL-6 and IL-8, respectively. Data shown are geometric means±SEM (n=18 subjects per treatment group). Data analysed with two-sample t-test. *P<0.05; **P<0.01 compared with placebo.

Pretreatment with GSK1995057 was associated with a reduction in the post-LPS BALF neutrophil count compared with placebo (figure 4A). This reduction became statistically significant when a single biological outlier who received GSK1995057 and exhibited an exaggerated increase in BALF neutrophils (>3 times IQR, and outside the upper quartile) was excluded (41% reduction, P<0.05 vs placebo; figure 4B). BALF cytology in this subject was abnormal with the presence of foamy macrophages, excessive mucus and debris, and lytic/apoptotic neutrophils; however, the subject was clinically well other than a transient fever (38.2°**C**) 13 and 14 hours after administration of LPS and GSK1995057, respectively, which was considered consistent with the expected clinical effects of LPS challenge. The BALF neutrophil data are presented both with and without this biological outlier. GSK1995057 had no effect on the numbers of macrophages or lymphocytes within the BALF at 6 hours (data not shown).

In keeping with our in vitro and animal model studies, GSK1995057 significantly reduced the BALF concentrations of IL-1β, IL-6 and IL-8 following LPS administration (P<0.01 in all cases; figure 4D–F), as well as reducing BALF concentrations of the mononuclear cell chemokines MIP1α, MIP1β and MCP-1 (P<0.001 in all cases; online supplementary figure 9A–C). Furthermore, GSK1995057 also reduced the BALF concentration of vWF after LPS challenge (P<0.05; figure 4C). This occurred in the absence of alterations in the BALF concentrations of the alveolar epithelial injury/activation markers SP-D and CC16; data not shown. These data again support the concept that GSK1995057 reduces pulmonary inflammation via modulation of neutrophil–endothelial interactions. We observed no differences in the BALF concentrations of the alveolar-capillary barrier permeability marker alpha-2-macroglobulin between placebo and GSK1995057-treated subjects (data not shown). Of note, however, the dose of LPS used in the human clinical trial was much lower than that used in the animal study where amelioration of LPS-induced permeability by GSK1995057 was observed (50 µg in humans vs 400 µg in animals). The increase in serum C-reactive protein 24 hours after LPS challenge was significantly attenuated by pretreatment with GSK1995057 (P<0.001; online supplementary figure 10), which was consistent with effects on the systemic acute phase response. Pharmacokinetic data demonstrated that inhaled doses of GSK1995057 reached the lung and subsequently, to a lesser extent, the systemic compartment (online supplementary figure 6).

## Discussion

Here we demonstrate that GSK1995057 reduced pulmonary inflammation in non-human primate and human models of ARDS, and confirm a mechanistic link between TNFR1 signalling and neutrophil/endothelial interactions. The observation that GSK1995057 significantly blunted neutrophil migration through endothelial monolayers in vitro suggests an important role for TNFR1 in mediating neutrophil–endothelial interactions, possibly by regulating the cell surface expression of endothelial cell adhesion molecules E-selectin, ICAM and VCAM. In patients with ARDS, neutrophil infiltration into the lungs is further augmented by a compromised alveolar-capillary barrier brought about by the action of proinflammatory mediators.^32^ Consistent with previous reports,^17 18 33^ we showed that TNF-α contributed to increased endothelial permeability and found that GSK1995057 prevented this increase, suggesting that TNFR1 signalling (rather than TNFR2 signalling) mediates TNF-induced endothelial permeability.

In some patients with ARDS, it may be sufficient to deliver short-acting therapeutics directly to the lungs; however, factors influencing the pulmonary delivery and disposition of antibodies or antibody fragments are complex,^34^ with limited precedence in humans. We therefore sought to determine whether nebulised GSK1995057 could be delivered to lungs efficiently, and whether the effects observed in in vitro assays could be recapitulated in vivo. To test this, we used the well-established model of lung LPS challenge to trigger a clinically relevant and complex inflammatory response to model subclinical tissue injury.^35–37^ In monkeys exposed to a single inhaled LPS challenge, pretreatment with nebulised GSK1995057 significantly reduced pulmonary neutrophil infiltration, levels of proinflammatory chemokines, markers of endothelial injury and alveolar-capillary leak in a dose-dependent fashion. These data are consistent with a single report of the effects of full IgG anti-TNF-α monoclonal antibodies in similar non-human primate models,^38^ and suggest an important role for TNFR1 signalling in the control of neutrophilic inflammation in vivo. Moreover, the data suggest that like other inhaled antibody approaches, low doses of inhaled GSK1995057 may deliver the same or more potent effects in the lung as higher doses of parenterally administered antibodies.^39^ Finally, we sought to translate our findings with GSK1995057 to human subjects through the conduct of a clinical trial in which healthy subjects were exposed to a low dose of inhaled LPS following a single nebulised dose of GSK1995057. Consistent with our investigations in monkeys, healthy subjects pretreated with GSK1995057 experienced less neutrophilic lung inflammation, and signs of endothelial injury in response to LPS challenge than subjects who received placebo. Subjects pretreated with GSK1995057 also showed signs of reduced systemic inflammation. Taken together, these studies highlight the therapeutic potential of GSK1995057, and the involvement of TNFR1 signalling in inflammation and tissue injury responses in primates and humans.

The current study has limitations. While BAL protein increased 24 hours after LPS challenge in monkeys, and GSK1995057 pretreatment attenuated this response, a similar GSK1995057 treatment effect was not apparent in human BAL taken 6 hours after LPS challenge in the clinical trial. BAL protein levels were not significantly increased in humans at 6 hours after LPS challenge, suggesting limited alveolar-capillary leak in response to pulmonary LPS challenge at this early time point. We were unable to assess the contribution of TNFR2 signalling in these studies, primarily due to a paucity of specific biomarkers associated with the TNFR2 pathway. Although we were unable to include a non-selective/pan-TNF-α inhibitor antibody as a comparator in our clinical trial, the observed effects of GSK1995057 on neutrophilic infiltration appear similar to the effects of the long-acting anti-TNF-α antibody adalimumab in the same model.^40^ Although neutrophil infiltration is a hallmark of ARDS pathophysiology, neutrophils are also likely to be important contributors to lung immunity, particularly in infectious causes of ARDS, and therefore the impact of therapies that modulate neutrophilia will need to be assessed carefully, in a stepwise manner. This small trial also only investigated a single, relatively high dose of GSK1995057 in male subjects. Further work to explore dose–response relationships in both men and women is required to support generalisability. However, data from the investigation in monkeys suggest GSK199507 may be efficacious at much lower nebulised doses, further supporting the utility of low doses of inhaled dAbs for the treatment of pulmonary diseases. Finally, in our studies, GSK1995057 was administered prior to the LPS challenge, which may impact the translation of our findings to patients since most patients with ARDS would be treated after the initial injury. However, at this early stage of investigation, confirming primary pharmacology of this novel selective TNFR1 antagonist in a clinically relevant model was an important step in determining the potential of this therapy in ARDS. Moreover, since the evolution of ARDS is often predictable,^41^ and ongoing injury, for example, at the onset of mechanical ventilation, is also likely to occur,^42 43^ our studies support the potential utility of GSK1995057 for prophylaxis or early treatment of ARDS.

In summary, our data suggest that selective antagonism of TNFR1 using an inhaled dAb may offer therapeutic benefit in patients with ARDS, a common and devastating condition that currently has no effective disease-modifying therapy. While the clinical effectiveness of this approach in established disease is yet to be tested, a phase IIa clinical trial of a TNFR1-targeting dAb in patients is currently under way.
