# Supplementary material for: Novel anti-tumour necrosis factor receptor-1 (TNFR1) domain antibody prevents pulmonary inflammation in experimental acute lung injury
Source: Thorax. 2018 Jan 29;73(8):723–30. doi: 10.1136/thoraxjnl-2017-210305 (PMC6204954; doi:10.1136/thoraxjnl-2017-210305)
Supplement: Supplementary file 1 [file thoraxjnl-2017-210305supp001.pdf]

Online Data Supplement

A novel anti-tumor necrosis factor receptor-1 (TNFR1) domain antibody prevents pulmonary inflammation in experimental acute lung injury

Alastair Proudfoot[1†], Andrew Bayliffe[2†,] Cecilia O'Kane[3†], Tracey Wright[2], Adrian Serone[4], Philippe Bareille[2], Vanessa Brown[3], Umar Hamid[3], Younan Chen[4], Robert Wilson[2], Joanna Cordy[2], Peter Morley[2], Ruud de Wildt[2], Stuart Elborn[3], Matthew Hind[1,5], Edwin Chilvers[6], Mark Griffiths[1,5‡], Charlotte Summers[6‡], Danny McAuley[3‡*]

Detailed Methods

In vitro studies in human lung cells

TNFα and GSK1995057 concentrations were selected based on *in-vitro* dose response experiments using IL-8 release following TNFα stimulation in alveolar type 2 cells. This data was found to be equivalent to previous data generated by GSK. A concentration of 10ng/mL TNFα (~EC50) and 10nM GSK1995057 (~IC90) was selected for the following experiments. IL-8 concentration was selected from a dose response experiment to optimise neutrophil migration through HMVEC-L monoayers.

Human microvascular endothelial monolayer permeability

The permeability of human pulmonary microvascular endothelial cell (HMVEC-L) monolayers was measured using an electrical cell impedance sensor technique (ECIS: Applied Biophysics, Inc., Troy, NY). HMVEC-L (2.5 x 105 cells/mL, 400 µL EGM-MV2 medium Promocell, Heidelberg, Germany, supplemented with 2% FBS, growth factors and antibiotics as per manufacturers recommendations) were cultured in proprietary gold electrode chamber slides (Applied Biosystems Inc, NY, USA). The chamber slide was connected to the 16-well array station and placed in an incubator at 37°C, 5% CO_2. Following growth to confluence, a 4,000-Hz AC signal with 1V amplitude through a 1-mH resistor created a constant current source (1 µA). Transendothelial resistance (TER) increased immediately after

cell attachment and achieved a steady state when endothelial cells became confluent (72 hours). Thus, experiments were conducted after the electrical resistance achieved a steady state of 3000 ohms. Resistance data were normalized to the initial TER (ohms) and recorded over 20 hours.

Neutrophil transmigration

To assess neutrophil migration through TNF-α stimulated HMVEC-L, a static migration assay (CytoSelect™ Leukocyte Transmigration assay, Cambridge Biosciences) was used. HMVEC-L (1.6 x105/mL, 200 μL EGM-MV2 medium supplemented with 2% FBS, growth factors and antibiotics as per manufacturers recommendations Promocell, Heidelberg, Germany) were seeded onto the upper chamber of Transwell® filters (24-well size, 3 μm pore size; Corning, USA) and left for 72 hours at 37°C, 5% CO_2. Prior to assessing neutrophil transmigration, growth to confluence and monolayer integrity was confirmed using phase contrast microscopy, TER measurement, confocal microscopy (to assess adherens junction formation in HMVEC-L grown on Transwell inserts), and FITC-labelled dextran exclusion. At 72 hours, confocal microscopy of Transwell filters stained strongly for VE-cadherin (anti-goat Alexafluor 594, Santa Cruz Biotech: data not shown) suggesting intact adherens junctions in monolayers. Transendothelial resistance increased over 72 hours, suggestive of monolayer integrity, and this effect was associated with lack of permeability to FITC-labelled albumin. After monolayer integrity was confirmed, Iscove's modified Dulbecco's medium (IMDM: 700 μL) was placed in the lower chamber. Cells were washed twice with PBS before the addition of GSK1995057 or dummy dAb (10nM) or vehicle control in medium for 1 hour prior to stimulation with TNFα (10ng/mL)for 4 hours at 37°C, 5% CO_2. All conditions were performed in triplicate. Following a further 2 washes in PBS (to remove basal chemokine release from stimulated HMVEC-L), the medium in the lower chamber was changed to IMDM containing IL-8 (50 ng/mL; R&D Systems). Peripheral blood neutrophils were isolated from healthy volunteers using dextran sedimentation and discontinuous plasma-Percoll® gradient centrifugation, as previously described (E1).

Neutrophils were suspended at 1x106 cell/mL in serum-free IMDM, and Leukotracker™ (Cambridge Bioscience, UK) added at the manufacturer's recommended dilution. Cells were incubated at 37°C, 5% CO_2 for 1 hour, washed twice and re-suspended at $1x10^6$ cells/mL in serum-free media; 200μL of this solution was added to the upper chamber, and neutrophils left to migrate for 3 hours. After 3 hours, the medium in the upper chamber was discarded and non-migratory cells removed with a cotton swab. The Transwell filter was then placed in a well containing lysis

buffer and medium from the lower chamber containing migrated neutrophils. The plate was gently shaken for 5 minutes at room temperature before the fluorescence was read, in triplicate, at 480 nm/520 nm.

Endothelial ligand cell surface expression

HMVEC-L were seeded into 6-well plates (1.2 x 10^5 cells/well) and grown to confluence over 3 days (37°C, 5% CO2). The effect of TNFR1 antagonism on adhesion molecule expression was assessed following 1 hour pre-incubation with GSK1995057 or dummy dAb (10nM) or vehicle control followed by TNFα (10ng/mL) stimulation for 4 hours. Cells were washed three times with Hanks Balanced Salt Solution, and harvested using non-enzymatic cell dissociation buffer (Sigma-Aldrich). The cell suspension was placed on ice and centrifuged at 400 g (5 minutes, 4°C). The supernatant was removed and the cell pellet re-suspended in blocking buffer (5% fetal bovine serum, 0.5% bovine serum albumin in PBS) for 10 minutes at 4°C. Antibodies to E-selectin (clone HCD62E, Biolegend), VCAM-1 (Clone STA, Biolegend) and ICAM-1 (Clone HA58, Biolegend), along with appropriate isotype and unstained controls were added and the cell incubated in the dark (30 minutes, 4°C). Cells were washed twice and re-suspended in stain buffer (PBS +1% FBS + 0.09% sodium azide) before being transferred to FACS tubes. Samples were analyzed on a FACSCanto™ II flow cytometer (BD Biosciences).

Preclinical study in cynomolgus monkeys

Study Design & Procedures

Adult male Cynomolgus monkeys weighing between 3.6-5.7kg, were chosen for this study because it is a species that has shown pharmacologic responses to GSK1995057, and is commonly used for efficacy studies in the LPS-challenge model. Determination of group size was based on feasibility but with a target of including at least *n*=5 animals per treatment group based on an assessment of the variability of planned BAL endpoints from historical data in this model. Animals were assigned to treatment groups by the study director based on baseline response to LPS in Session 1 to ensure a balance of LPS responsiveness across treatment groups following the design schematic below:

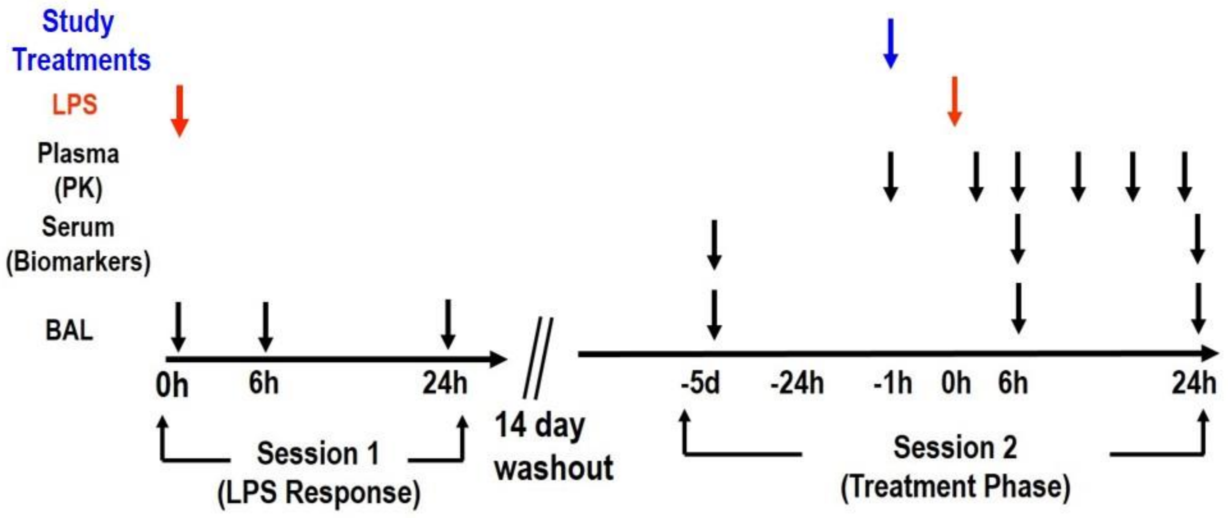

Sedation was induced with intramuscular ketamine (5-10 mg/kg) to allow placement of a venous catheter, and induction of anesthesia with a bolus of intravenous propofol (3mg/kg). Anesthesia was maintained with a continuous intravenous infusion of propofol (0.1-0.2 mg/kg/min). Treatments were administered 1 hour before LPS challenge. Group 1 was administered positive control (fluticasone propionate; 0.3 mg/kg); Group 2 was administered vehicle (20 mM phosphate buffer, pH 7.4, containing 4% poloxamer 124 and 0.01% polysorbate 80); and Groups 3, 4, and 5 were administered GSK1995057 at dose levels of 0.01, 0.1 and 1 mg/kg, respectively. For Group 1, the positive control was delivered via intratracheal dosing by guiding a pediatric fiberoptic bronchoscope past the carina to wedge in a major bronchus. Doses were administered at 0.3 mg/kg using a dose volume of 0.5 mL/kg per lung (total of 1 mL/kg per animal). Doses were rounded to the nearest 0.1 mL, with the exception of Animal Nos. 1001 and 1002 that were rounded to the nearest 1.0 mL. This rounding error resulted in Animal No. 1001 receiving 0.2 mL less and Animal No. 1002 receiving 0.3 mL more of fluticasone propionate than intended.

The doses of vehicle (Group 2) and GSK1995057 (Groups 3–5) were delivered by aerosol inhalation using a Pari LC jet nebulizer (Pari, USA) at a dose volume of 0.5 mL/kg. Each treatment was delivered at a calculated dose time determined for each animal based on body weight. Therefore, based on mean body weights at the time of dosing, the total delivered doses (i.e. nominal doses) in this study were 1.2mg of fluticasone propionate in Group 1, and 0.043mg, 0.45mg, and 4.7mg of GSK1995057 in Groups 3, 4, and 5, respectively. Dose times ranged between 9 and

14.25 minutes and an additional 3.5 mL of the dosing formulation was added to the nebulizer chamber for each dose to compensate for dead space and possible foaming during the nebulisation.

Bronchoalveolar lavage

A paediatric fiberoptic bronchoscope was wedged into a segmental bronchus, and 3 aliquots of sterile 0.9% saline (20 mL) instilled. Bronchoalveolar lavage return was measured, and samples placed on ice until transferred to the laboratory for processing. BALF was centrifuged and the supernatant removed and stored at –80°C for subsequent analysis. The cell pellet was re-suspended in phosphate buffered saline (2 mL). Cytospins were prepared and stained with Wright-Giemsa, before a minimum of 200 nucleated cells were counted to determine the differential cell counts.

Clinical trial in healthy volunteers

Subjects

Healthy subjects were recruited by advertising. Screening consisted of a history and physical examination, blood investigations, ECG, and spirometry (the full inclusion and exclusion criteria and study schedule are outlined in Supplementary Table E1 and Supplemental data E1).

Study Design

The study was a randomized, placebo-controlled clinical trial. The participants, site investigators and the staff performing the laboratory assays were blinded to the treatment allocation. The site clinical trials pharmacist and central GSK study team were unblinded to treatment allocation. Eligible subjects received a unique subject number. Subjects were assigned to receive a total nebulised dose of GSK1995057 (26 mg) or identical placebo (1:1) in accordance with the randomization schedule generated by Clinical Pharmacology and Biometrics at GSK. When an eligible subject was recruited, the clinical trials pharmacist allocated the subject to the designated treatment group, maintaining blinding. Details of the study medication are given in Supplementary table 2. The nebulizer solutions had an identical appearance. Study drug was administered using a Pari eFlow nebuliser. One hour after the study medicine was taken, subjects inhaled LPS (total dose 50 μg) using a large-volume reservoir delivery system as described (E2). BAL was performed 6 hours after LPS inhalation (7 hours after dosing GSK1995057). Blood was collected as defined in the schedule of events in the online supplement (Supplementary Table 1). LPS inhalation (Escherichia coli serotype O26:B6; Sigma Chemicals, Poole, Dorset, UK) was dissolved in endotoxin free sterile

0.9% saline and inhaled via an automatic inhalation–synchronized dosimeter nebuliser (Spira, Hameenlinna, Finland). This delivers particles of a mass median aerodynamic diameter (MMAD) of 10 µm as described previously (E3). The dosimeter produces a calibrated aerosol of 8 µL at each slow inhalation starting from functional residual capacity (FRC) to total lung capacity (TLC). Each subject performed five successive inhalations of the LPS solution (1.25 mg/mL) through the mouthpiece with a nose clip in place. The total dose of inhaled LPS was 50 µg.

The BAL procedures, as well as the processing of BALF and blood, were performed as described below. Subjects stayed overnight in a clinical research unit following dosing with LPS.

Bronchoalveolar lavage

Bronchoalveolar lavage was performed 6 hours after LPS inhalation according to standard guidelines (E4). Three successive 60 mL aliquots of 0.9% saline were instilled into a sub-segment of the right middle lobe and each aspirated immediately with low suction. Bronchoalveolar lavage fluid (BALF) return was measured and immediately placed on ice until transferred to the laboratory for processing. BALF was centrifuged at 900g for 5 minutes at 4°C. The supernatant was removed and stored at –80°C for subsequent analysis. The cell pellet was re-suspended in phosphate buffered saline (PBS: 10 mL) supplemented with 1% bovine serum albumin (BSA) (Sigma Chemicals). Total cell count was determined using a hemocytometer. Cell viability was measured by the ability of live cells to exclude Trypan blue. Cytospins were prepared and stained with Speedy-Diff (Clin-Tech Ltd, UK). A single cytologist who was blinded to treatment allocation performed the differential cell counts. Epithelial lining fluid (ELF) concentrations of GSK1995057 were derived by normalising to concentrations of ELF urea (E5).

Plasma samples

Blood was collected into K3 EDTA tubes, mixed gently, and placed immediately on ice until processed. Blood was centrifuged at 1500g for 10 minutes at 4°C. The plasma was removed and stored at -80°C for subsequent analysis.

Serum Samples

Blood was collected into serum separator tubes, mixed gently and maintained at ambient temperature for 30 minutes to 1 hour to allow for complete clotting before centrifugation at 1500g for 15 minutes at 4°C. The serum was removed and stored at -80°C for subsequent analysis.

This GSK clinical trial (GSK study ID: 116236) was prospectively registered on ClinicalTrials.gov (NCT01587807) and the clinical trial protocol and results summaries are available at the GSK clinical trial registry website (https://www.gsk-clinicalstudyregister.com).

Supplement References:

E1. Haslett C, Guthrie LA, Kopaniak MM, Johnston RB, Henson PM. Modulation of multiple neutrophil functions by preparative methods or trace concentrations of bacterial lipopolysaccharide. *Am. J. Pathol*. **119**, 101-101 (1985).

E2. Shyamsundar M, McKeown STW, O'Kane CM, Craig TR, Brown V, Thickett DR, Matthay MA, Taggart CC, Backman JT, Elborn JS, McAuley DF. Simvastatin decreases lipopolysaccharide-induced pulmonary inflammation in healthy volunteers. *Am. J. Respir. Crit. Care Med*. **179**, 1107-1114 (2009).

E3. Sohy C, Pons F, Casset A, Chesnard MP, Lieutier-Colas F, Meyer P, Pauli G, de Blay F. Low-dose endotoxin in allergic asthmatics: effect on bronchial and inflammatory response to cat allergen. *Clin. Exp. Allergy* **36**, 795-802 (2006).

E4. British Thoracic Society Bronchoscopy Guidelines Committee. British Thoracic Society guidelines on diagnostic flexible bronchoscopy. *Thorax* **56**, i1-i21 (2001).

E5: Rennard et al., Estimation of volume of epithelial lining fluid recovered by lavage using urea as a marker of dilution. *J Appl. Physiol*. **60** (2). 532-538. (1986).

Figure Legends

Figure. E1. Homology model structure and amino acid sequence of TNFR1 domain antibody, and binding of TNFR1 domain antibody fragment to human and Cynomolgus monkey TNFR1:Fc, measured by surface plasmon resonance.

Figure. E2. The effect of GSK1995057 on the release of endothelial activation/injury markers from TNF-α stimulated HMVEC-L monolayers.

Figure. E3. Effect of GSK1995057 on the release of IL-1β, IL-6 and IL-8 from TNF-α stimulated HMVEC-L monolayers.

Figure. E4. Effect of pre-treatment with GSK1995057 prior to LPS-inhalation on BALF myeloperoxidase concentrations and macrophage counts in Cynomolgus monkeys.

Figure. E5. Schematic of dose escalation in clinical study.

Figure. E6. Median plasma GSK1995057 concentration profiles over 48 hours following administration of escalating single inhaled doses of GSK1995057 given during the dose escalation phase of the clinical trial (Part 1; Cohorts 1-4).

Figure. E7. Concentrations of free and total soluble TNFR1 in BALF after treatment with GSK1995057 or vehicle control from Cohort 6 in the LPS challenge part (Part 2) of the clinical trial.

Figure. E8. Schematic of double blind placebo controlled clinical trial.

Figure. E9. Effect of pre-treatment with GSK1995057 on BALF concentrations of MIP-1α, MIP-1β and MCP-1 in healthy volunteers exposed to inhaled LPS (Part 2; Cohort 6)

Figure. E10. Effect of pre-treatment with GSK1995057 on C-reactive protein (CRP) concentrations in the serum of healthy volunteers exposed to LPS (Part 2; Cohort 6).

Table E1. Schedule of clinical trial events.

Table E2. Investigational product and placebo for clinical trial.

Table E3. Adverse events in clinical trial.

Data file E1. Clinical trial inclusion and exclusion criteria.

Supplementary Figure 1

(A)

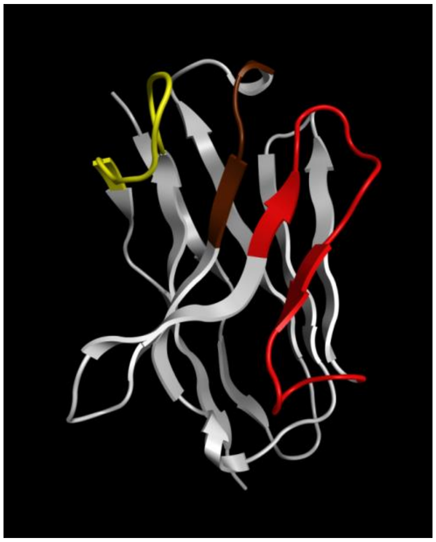

Amino acid sequence:
EVQLLESGGGLVQPGGSLRLSCAASGFTFAHETMVWVRQAPGKGLEWVSHIPPDGQDPFYADSV
KGRFTISRDNSKNTLYLQMNSLRAEDTAVYHCALLPKRGPWFDYWGQGTLVTVSS

Panel (**A**) shows the Kabat defined CDRs: CDRH1 = dark red; CDRH2 = red; CDRH3 = yellow.

(B)

	**KD (nM)**
**Human**	0.014
**Cynomolgus monkey**	0.015

Supplementary Fig 1: (A) Homology model structure and amino acid sequence of TNFR1 domain antibody (B) Binding of TNFR1 domain antibody fragment to human and Cynomolgus monkey TNFR1:Fc, measured by surface plasmon resonance

Supplementary Figure 2

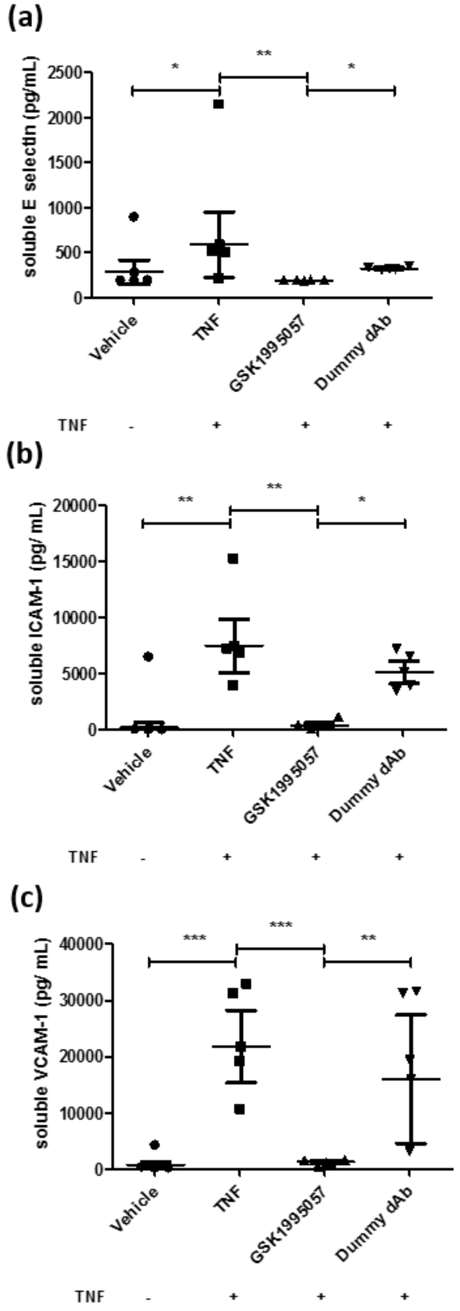

Supplementary Fig 2: The effect of GSK1995057 on the release of endothelial activation/injury markers from TNF-α stimulated HMVEC-L monolayers.

N=4 for all groups. * indicates $P<0.05$, ** indicates $P<0.01$, *** indicates $P<0.001$

Supplementary Figure 3

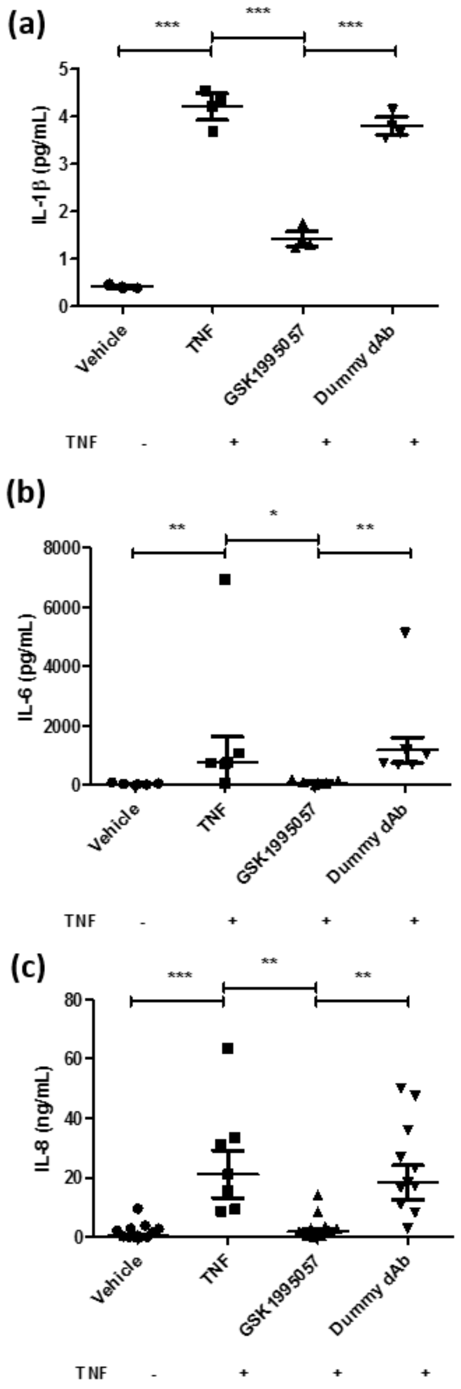

Supplementary Fig 3: Effect of GSK1995057 on the release of IL-1β, IL-6 and IL-8 from TNF-α stimulated HMVEC-L monolayers.

N=3-11 for all groups. * indicates $P<0.05$, ** indicates $P<0.01$, *** indicates $P<0.001$

Supplementary Figure 4

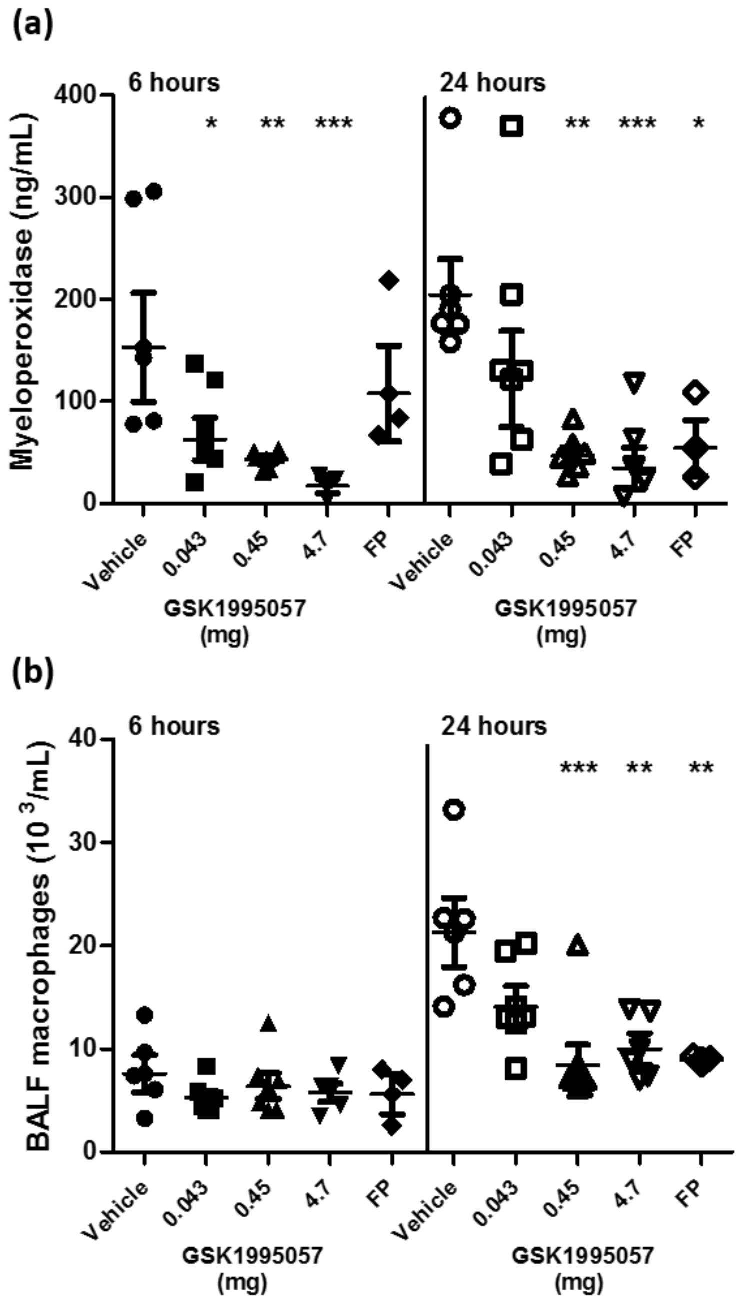

Supplementary Fig 4: Effect of pretreatment with GSK1995057 prior to LPS-inhalation on BALF myeloperoxidase concentrations and macrophage counts in cynomolgus monkeys.

* indicates *P*<0.05, ** indicates *P*<0.01, *** indicates *P*<0.001. FP indicates fluticasone propionate.

Supplementary Figure 5

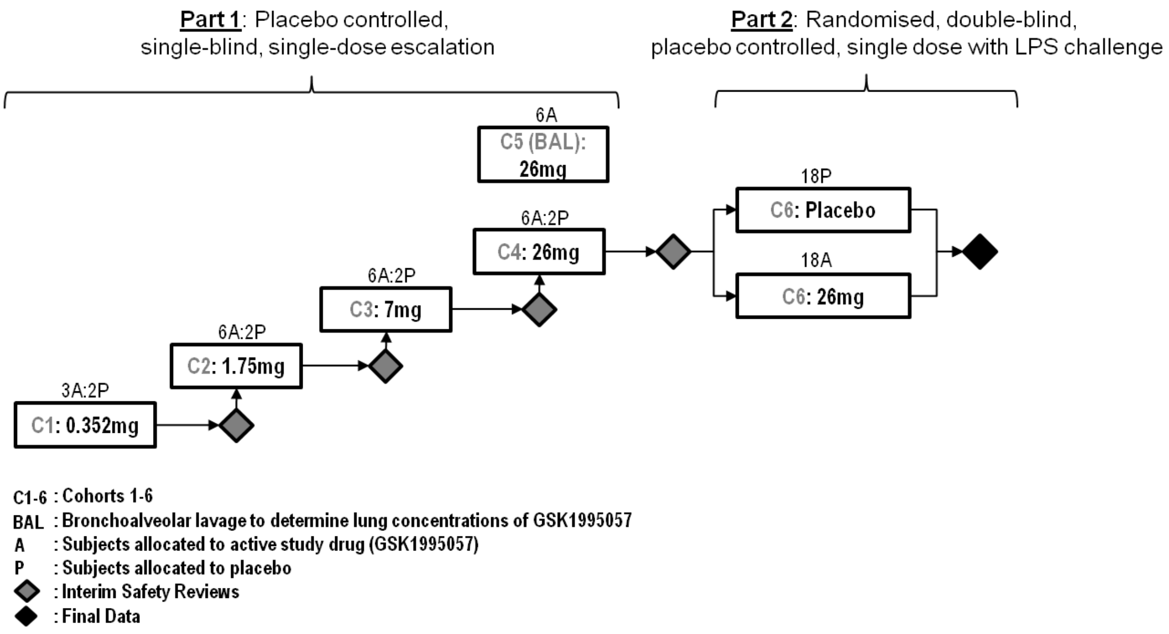

Supplementary figure 5: Schematic of dose escalation in clinical study.

Supplementary Figure 6

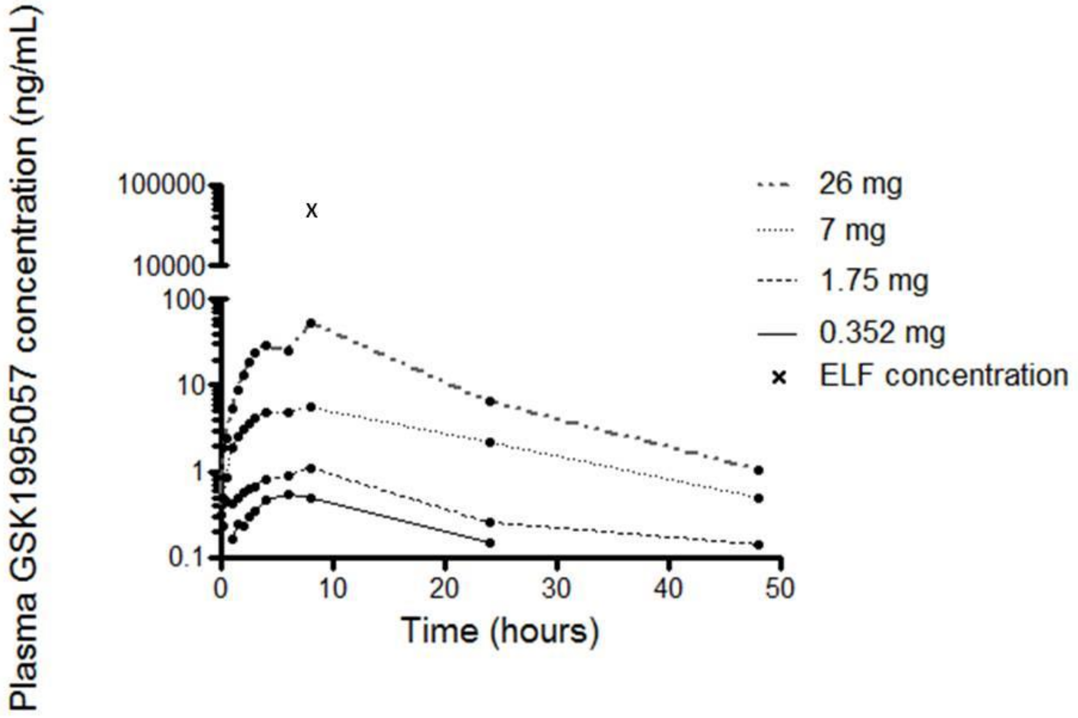

Supplementary Fig 6: Median plasma GSK1995057 concentration profiles over 48 hours following administration of escalating single inhaled doses of GSK1995057 given during the dose escalation phase of the clinical trial (Part 1; Cohorts 1-4).

Median Lung epithelial lining fluid (ELF) GSK1995057 concentrations also assessed in BALF samples taken 7 hours following administration of a single 26 mg inhaled dose of GSK1995057 to subjects in Cohort 6 (LPS challenge).

Supplementary Figure 7

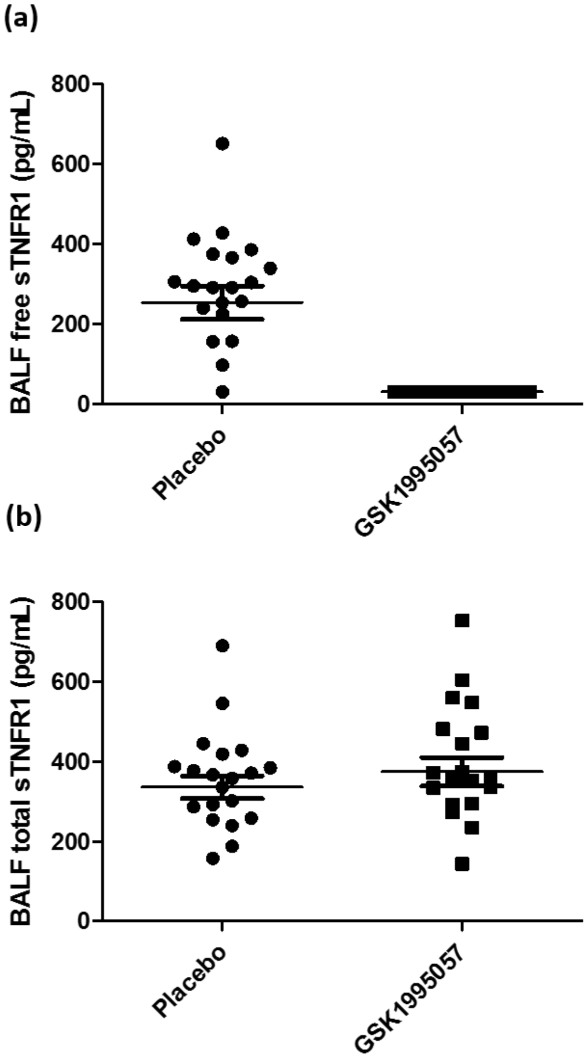

Supplementary Fig 7: Concentrations of free and total soluble TNFR1 in BALF after treatment with GSK1995057 or vehicle control from Cohort 6 in the LPS challenge part (Part 2) of the clinical trial.

Free and total sTNFR1 were measured to monitor the interaction of GSK1995057 with sTNFR1. The assay for free sTNFR1 detects sTNFR1 not bound to GSK1995057, whereas the assay for total sTNFR1 detects both free sTNFR1 and sTNFR1 bound to GSK1995057. No error bar shown in panel (a) for GSK1995057 group as all values measured were below the lower limit of quantification of the assay (61.73 pg/mL).

Supplementary Figure 8

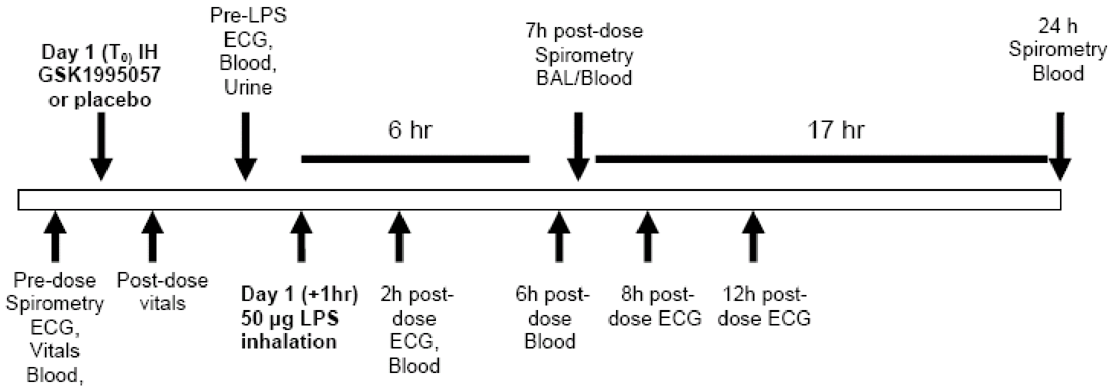

Supplementary Fig 8: Schematic of double blind placebo controlled clinical trial.

Supplementary Figure 9

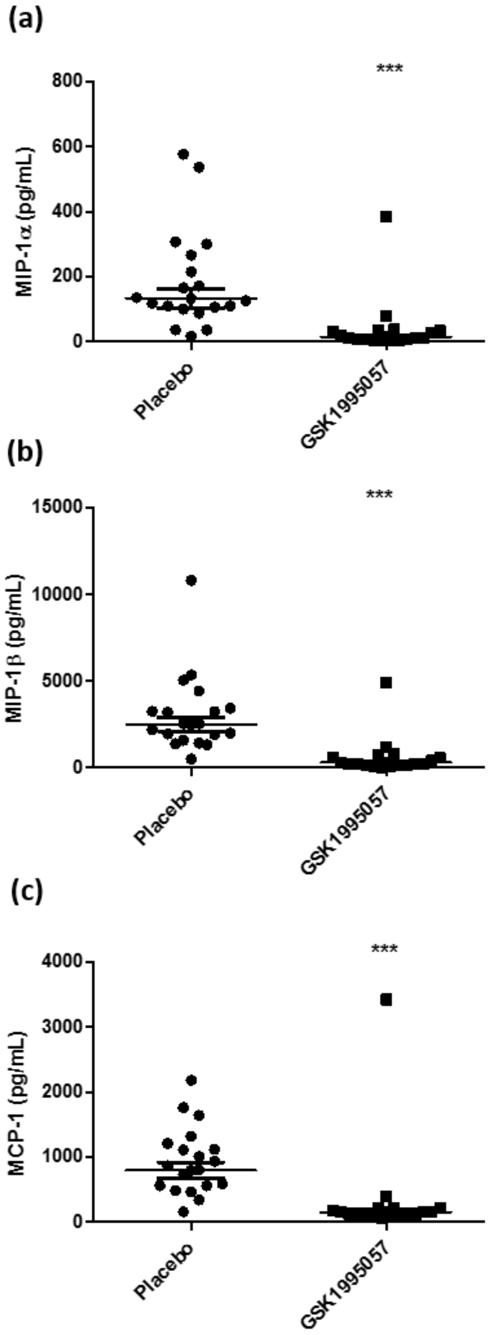

Supplementary figure 9: Effect of pretreatment with GSK1995057 on BALF concentrations of MIP-1α, MIP-1β and MCP-1 in healthy volunteers exposed to inhaled LPS (Part 2; Cohort6).

*** indicates *P*<0.001.

Supplementary Figure 10

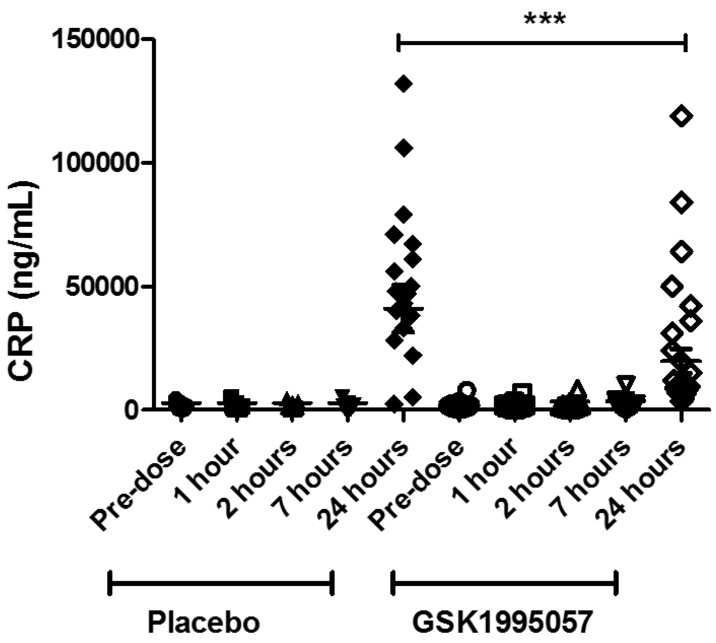

Supplementary Fig 10: Effect of pre-treatment with GSK1995057 on C-reactive protein (CRP) concentrations in the serum of healthy volunteers exposed to LPS (Part 2; Cohort 6).

*** indicates $P<0.001$.

Supplementary Table 1: Schedule of clinical trial events.

Procedure	Screening	Study Day										F/U
	Up to 28 days prior to Day 1	D1								D2	D7	D29 +/-5
		Pre-dose	0 h	+1 h	+2 h	+6 h	+7 h	+8 h	+12 h	+24 h		
Informed Consent	X											
Demographics	X											
Eligibility	X											
Full Physical Exam	X											
Brief Physical Exam		X										X
Medical/Medication/ Drug/Alcohol History	X											
12-lead ECG	X	X			X				X		X	X
Holter Monitoring	X											
Vital Signs	X	X								X	X	X
Lead II Cardiac Telemetry		X	X	X	X							
Spirometry (FEV1, FVC)	X	X					X			X		
Urine Drug/Alcohol	X	X										
Serum β-hCG test (♀)	X											X
Urine Pregnancy Test (♀)		X										
HIV, Hep B and C Screen	X											
Haem/Chem/Urinalysis Tests	X	X		X						X	X	X
Influenza Screen	X											
QuantiFERON Gold TB Test	X											
Toxoplasmosis Test	X											
Dosing			X									
LPS inhalation				X								
Bronchoscopy and BAL							X					
PK Blood Sampling		X			X	X	X			X		
Immunogenicity Sample	X										X	X
Biomarker Serum Sample		X		X	X		X			X		
Adverse Event Review	X	X								X	X	X
Con Medicines Review	X	X								X	X	X
Admission		X										
Discharge										X		
Outpatient Visit	X										X	X

Supplementary Table 2: Investigational product and placebo for clinical trial.

Product name:	GSK1995057	Placebo
Formulation description:	GSK1995057 formulated with sucrose, glycine, sodium dihydrogen phosphate and polysorbate 80	Sucrose, glycine, sodium dihydrogen phosphate and polysorbate 80
Dosage form:	Lyophilized solid for reconstitution prior to nebulization	Aqueous solution for nebulization
Unit dose/Dosage level:	26 mg	Volume to match active dose
Route/ Administration/ Duration:	Nebulized<br>Duration of nebulization will be approximately 2-5 min	To match active

Supplementary Table 3: Adverse events in clinical trial.

**Adverse event**	**Placebo (n=19)**	**GSK1995057 (n=18)**
Any adverse event	14 (74%)	11 (61%)
Any respiratory event (cough, chest pain, dyspnoea)	7 (37%)	7 (39%)
Headache	7 (37%)	5 (28%)
Fever (>37.8°C)	7 (37%)	3 (17%)
Nausea/vomiting	4 (21%)	0
White cell count increase	1 (5%)	0
Microscopic haematuria	2 (11%)	0

Supplementary Data File 1: Clinical trial inclusion and exclusion criteria.

Inclusion Criteria

1. Healthy as determined by a responsible and experienced physician, based on a medical evaluation including medical history, physical examination, laboratory tests and cardiac monitoring.
2. Between 18 and 55 years of age inclusive.
    a. A female subject was eligible to participate if she was of non-childbearing potential defined as pre-menopausal females with a documented tubal ligation or hysterectomy; or postmenopausal defined as 12 months of spontaneous amenorrhea.
    b. Male subjects agreed to use contraception from the time of the first dose of study medication until the last follow-up visit.
3. Normal creatinine clearance values at screening (calculated from serum creatinine by a predicting equation using Cockcroft-Gault formula), normal urine microscopy and no significant proteinuria on dipstick testing.
4. Body weight ≥ 50 kg and BMI within the range 19 – 29.9 kg/m^2 (inclusive).
5. No evidence of previous or active tuberculosis (TB) and a negative QuantiFERON TB Gold test taken within 28 days of dosing, and negative medical history with respect to active or latent mycobacterium tuberculosis complex infection.
6. Normal spirometry (FEV1 ≥ 85% of predicted, FEV1/FVC ratio ≥ 70%).
7. Capable of giving written informed consent.
8. Available to complete all study assessments.
9. Subjects who were able to use the inhaler device correctly.

10. Able to read, comprehend and write English at a sufficient level to complete study related materials.

Exclusion Criteria

A subject was not eligible for inclusion in this study if any of the following criteria applied:

1. A history of Hepatitis B, Hepatitis C or HIV infection and/or a positive pre-study HIV, Hepatitis B surface antigen or positive Hepatitis C antibody result within 3 months of screening.
2. Current or chronic history of liver disease, or known hepatic or biliary abnormalities.
3. A positive pre-study drug or alcohol screen.
4. History of and/or a positive test for toxoplasmosis consistent with active toxoplasmosis infection at the time of enrolment.
5. A positive RT-PCR test for influenza A/B.
6. Current evidence or history of an influenza-like illness as defined by fever (>38^0C) and two or more of the following symptoms within the last 7 days: cough, sore throat, runny nose, sneezing, limb/joint pain, headache, vomiting/diarrhoea in the absence of a known cause, other than influenza.
7. Corrected QT interval (QTc) >450msec.
8. History of regular alcohol consumption within 6 months of the study defined as an average weekly intake of >21 units for males or >14 units for females.
9. Subject unwilling to abstain from alcohol consumption from 24 hours prior to dosing until discharge from the clinic, and for 24 hours prior to all other out-patient clinic visits.
10. Current or previous smokers.
11. The subject has participated in a clinical trial and has received an investigational product within the following time period prior to the first dosing day in the current study: 90 days, 5 half-lives or

twice the duration of the biological effect of the investigational product (whichever is longer) or exposure to more than four new chemical entities within 12 months prior to the first dosing day.

12. Subjects having received any type of vaccination within 3 weeks of the anticipated dosing event or, are expected to be vaccinated, within 3 weeks post last dose.
13. Current evidence of ongoing or acute infection, history of repeated or chronic significant infections or history of serious infection within three months of randomization.
14. Subjects with asthma or a history of asthma outside childhood, COPD, other respiratory conditions or recurrent infections.
15. Use of prescription or non-prescription drugs (except simple analgesics), including vitamins, herbal and dietary supplements within 7 days or 5 half-lives (whichever is longer) prior to the first dose of study medication.
16. History of sensitivity to any of the study medications, or components thereof or a history of drug or other allergy including severe allergic reaction, angioedema or anaphylaxis.
17. History of malignancy, except for adequately treated non-invasive cancer of the skin (basal or squamous cell) or cervical carcinoma in situ (>2 years prior to dosing).
18. Where participation in the study would result in donation of blood or blood products in excess of 500 mL within a 3 month or 90 day period or if the subject has undergone a bone marrow donation within the last 6 months prior to screening.
19. Subject was unable to refrain from travelling to countries with a high prevalence of TB or other areas of prevalent infectious from the start of screening until the final follow-up visit.
20. Subject was mentally or legally incapacitated.
21. Subjects with a positive screening result for pre-existing GSK1995057 anti-drug antibodies.
